# Epileptiform Activity and Seizure Risk Follow Long‐Term Non‐Linear Attractor Dynamics

**DOI:** 10.1002/advs.202411829

**Published:** 2025-04-07

**Authors:** Richard E Rosch, Brittany Scheid, Kathryn A Davis, Brian Litt, Arian Ashourvan

**Affiliations:** ^1^ Departments of Pediatrics and Neurology Columbia University Irving Medical Center New York NY 10032 USA; ^2^ Department of Basic and Clinical Neuroscience Institute of Psychiatry Psychology and Neuroscience King's College London Cutcombe Road London SE5 9RT UK; ^3^ Department of Bioengineering School of Engineering and Applied Science University of Pennsylvania Philadelphia PA 19104 USA; ^4^ Penn Center for Neuroengineering and Therapeutics University of Pennsylvania Philadelphia PA 19104 USA; ^5^ Department of Neurology Perelman School of Medicine University of Pennsylvania Philadelphia PA 19104 USA; ^6^ Department of Psychology University of Kansas Lawrence 66045 USA

**Keywords:** delay‐embedding, Hankel alternative view of Koopman (HAVOK), singular value decomposition (SVD)

## Abstract

Many biological systems display circadian and slow multi‐day rhythms, such as hormonal and cardiac cycles. In patients with epilepsy, these cycles also manifest as slow cyclical fluctuations in seizure propensity. However, such fluctuations in symptoms are consequences of the complex interactions between the underlying physiological, pathophysiological, and external causes. Therefore, identifying an accurate model of the underlying system that governs the multi‐day rhythms allows for a more reliable seizure risk forecast and targeted interventions. The primary aim is to develop a personalized strategy for inferring long‐term trajectories of epileptiform activity and, consequently, seizure risk for individual patients undergoing long‐term ECoG sampling via implantable neurostimulation devices. To achieve this goal, the Hankel alternative view of Koopman (HAVOK) analysis is adopted to approximate a linear representation of nonlinear seizure propensity dynamics. The HAVOK framework leverages Koopman theory and delay‐embedding to decompose chaotic dynamics into a linear system of leading delay‐embedded coordinates driven by the low‐energy coordinate (i.e., forcing). The findings reveal the topology of attractors underlying multi‐day seizure cycles, showing that seizures tend to occur in regions of the manifold with strongly nonlinear dynamics. Moreover, it is demonstrated that the identified system driven by forcings with short periods up to a few days accurately predicts patients' slower multi‐day rhythms, which improves seizure risk forecasting.

## Graphical abstract

This study leverages the HAVOK framework to model long‐term, nonlinear attractor dynamics underlying epileptiform activity and seizure risk in epilepsy patients. By identifying key forcing mechanisms driving chaotic transitions, the findings improve seizure risk forecasting over multi‐day cycles and provide a pathway for personalized, data‐driven interventions in epilepsy management.

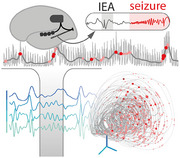

## 1 Introduction

Many biological processes exhibit endogenous cyclic behavior at different time scales, ranging from faster circadian rhythms to slower weekly or monthly cycles.^[^ ^1^, ^2^ ^]^ Circadian rhythms, which repeat every 24 h, are observed in several physiological processes, such as sleep cycles and gene expression patterns.^[^ ^2^ ^]^ Similarly, slower multi‐day cycles are observed, including cardiac^[^ ^3^ ^]^ and menstrual cycles.^[^ ^4^ ^]^ There have been long‐standing observations of temporal patterns at similar time scales in pathological processes across various disorders, including immunological and cardiological,^[^ ^5^, ^6^ ^]^ and mental health disorders.^[^ ^7^, ^8^ ^]^ Indeed, cyclic patterns were once considered so essential to certain paroxysmal disorders that their terminology reflects this. For example, the term “lunatic” originates from the belief that epileptic seizures were caused by lunar cycles.^[^ ^9^ ^]^ Seizure cycles have long been described and more recently validated quantitatively using seizure diary data^[^ ^10^ ^]^ and long‐term electroencephalographic recordings, e.g., from implantable neurostimulation devices such as Neuropace's Responsive Neurostimulator (RNS) device.^[^ ^11^, ^12^, ^13^, ^14^, ^15^ ^]^ In addition to seizures, other markers of pathophysiological cortical excitability, such as interictal epileptiform activity (IAE), have also shown multi‐day cycles. There is a reported phase relationship between cycles estimated from both physiological and pathophysiological measures, suggesting that both may reflect shared underlying causes.^[^ ^10^, ^16^ ^]^

Cyclical physiological patterns often emerge from internal ‘clock’ like mechanisms, further shaped by external input. For example, diurnal variation in the hypothalamic‐pituitary‐adrenal axis is powerfully entrained by light exposure^[^ ^17^ ^]^ but shows cyclical behavior even in the absence of external environmental cues. The relationship between internal drivers and external influences is less well understood for multi‐day cyclical patterns. While lunar entrainment of seizure cycles was once a hypothesis, a closer examination of RNS's interictal epileptiform activity (IEA) cycles and circular statistics has revealed no evidence supporting it.^[^ ^15^ ^]^

Despite our limited mechanistic understanding, multi‐day cycles have shown some promise in assessing and forecasting seizure risk for individual patients based on simple statistical descriptions of cyclical regularities.^[^ ^16^, ^18^ ^]^ Accurately forecasting seizure cycles from prior data over the long term is particularly challenging due to the irregular changes in the cycles themselves over time, which are not well captured by existing modeling approaches. These existing approaches effectively characterize fluctuations in epileptiform activity statistically but do not provide a systematic framework for reconstructing the underlying dynamical structure over extended time scales. This limits our current ability to provide more accurate forecasts or identify markers associated with significant changes in dynamical regimes. To address this, we aimed to apply a data‐driven framework that reconstructs complex attractors from empirical time series, allowing us to identify and forecast transitions in the dynamic regime, and identify early markers of impending changes.Therefore, developing a principled framework for generative modeling of these seemingly chaotic dynamics that go beyond statistical descriptions of limited observed data could improve our understanding of the underlying causes and enable more reliable forecasts of impending seizure events.

Modeling time‐varying expression of pathophysiology using a dynamical systems framework has been used to represent, e.g., dynamic patterns observed in psychopathology.^[^ ^19^ ^]^ Such a framework offers model‐based predictions and simulations of control‐theoretic interventions, such as drugs or neuromodulation therapy. However, the approach relies on prior knowledge about the system's underlying dimensions and equations, which are typically not known for complex dynamical pathophysiology such as epilepsy.

Recent advances in data‐driven system identification methods, such as the sparse identification of nonlinear dynamical systems (SINDy),^[^ ^20^ ^]^ provide a reliable tool for discovering the equations and normal forms^[^ ^21^ ^]^ of real‐world dynamics. Such simplified representations of the high‐dimensional nonlinear or chaotic dynamics are beneficial, as they provide valuable information about the system's important macroscopic behavior, such as the emergence of bifurcations^[^ ^22^ ^]^ or switching phenomena.^[^ ^23^ ^]^

The occurrence of individual seizures is likely caused by multiple factors, reflecting the dynamics of the high‐dimensional system of physiological, pathophysiological, and environmental causes. The temporal profile of seizure risk can be approximated with some accuracy from individual physiological measurements,^[^ ^10^, ^18^ ^]^ but these do not reflect the complexity of the full underlying dynamics. However, we can reconstruct a diffeomorphic attractor from a single measurement to the underlying measured attractor based on Taken's seminal embedding theorem.^[^ ^24^ ^]^ Taken's theorem implies that the attractors' full dynamics and important information regarding their topology can be uncovered by delay‐embedding a single relevant measurement. More recently, a framework combining the delay‐embedded coordinates and dynamic mode decomposition – Hankel Alternative View of Koopman (HAVOK) – was shown to allow the decomposition of nonlinear chaotic dynamics into a linear dynamical system with intermittent forcing captured by the low‐energy delay coordinates.^[^ ^23^ ^]^ Increases in forcing were shown to mark periods with high nonlinearity followed by switching and bursting behavior in silico, as well as in real‐world examples. For instance, increased forcing predicts the switching between the lobes in the Lorenz attractor, changes in the earth's magnetic field, and measles outbreaks.^[^ ^23^ ^]^

In this study, we utilized the delay‐embedding approach to investigate the topology of attractors underlying multi‐day seizure cycles in patients with RNS implants. Our results indicate that a linear model driven by higher frequency forcing, using delay‐embedded coordinates, can accurately predict the future trajectory of IEA‐count time series as well as the occurrence of ‘long events’ (LEs). These are automatically detected seizure‐like electrocorticography patterns, the detection of which is individually tuned by the clinicians to reflect patient‐specific seizure patterns. Additionally, we observed that an increase in forcing drives the system into state‐space regions marked by non‐linear dynamics and increased seizure likelihood. Notably, our findings demonstrate that the forcing can accurately forecast impending seizures for up to several days in most patients, providing a data‐driven approach that offers a mechanistic view of the chaotic dynamics of seizure cycles. The model output may represent an accurate model‐based biomarker for impending seizures in patients undergoing long‐term electrophysiological monitoring.

## 2 Results

**Figure 1:**
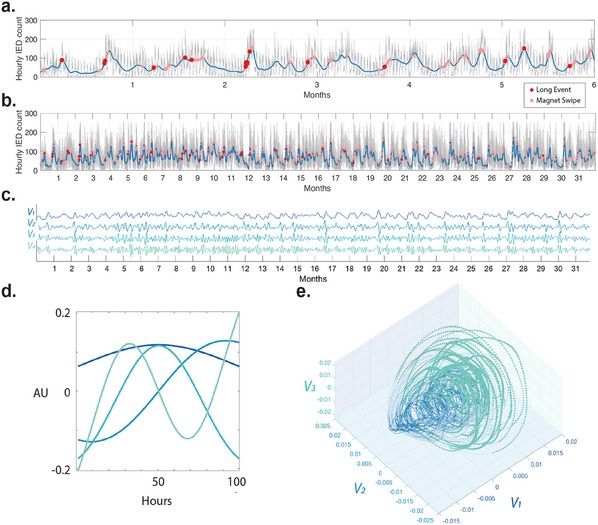
Delay‐embedded coordinates of IEA‐count time series reveal the topology of the attractor governing seizure risk. a) IEA hourly count time series from a representative patient. The blue line indicates the low‐pass (three‐day) filtered time series. Red dots show clinical seizures labeled by the patient using magnet swipes. Pink dots show the LEs detected by the RNS device, and their size indicates their hourly count. (b) Same as (a) over a longer window (32 months). c) The delay‐embedded coordinate time series (*V* matrix columns) of the average IEA curve in panel (b) calculated using Singular Value Decomposition (SVD) of the delay‐embedded matrix (i.e., Hankel matrix, see Experimental Section for more detail). d) The basis vectors (*U* matrix columns) corresponding to the delay‐embedded coordinate time series in panel (c). e) Reconstructed attractor. Samples with larger amplitude (measured using line length) of the higher frequency delay‐embedded coordinate (*V*_4_) oscillations are size‐ (amplitude) and color‐coded (*V*_4_'s normalized line length >0).

We hypothesized that the multi‐day cycles in hourly IEA counts of patients undergoing long‐term electrocorticography monitoring through an RNS device are the reflection of an underlying nonlinear system with chaotic dynamics. In order to uncover the topology of the underlying attractor, we first applied a low‐pass filter aiming to dampen circadian oscillations, with a three‐day cut‐off period to the hourly IEA‐count time series to reduce the power of the higher frequency circadian cycles (**Figure** **1**a,b) – see Experimental Section for more details on the rationale for low‐pass filtering. Next, we constructed a high‐dimensional (*d* = 100) delay‐embedded matrix (i.e., Hankel matrix) by stacking time‐shifted versions of the filtered hourly IEA‐count time series. The delay‐embedded matrix's singular value decomposition (SVD) allows us to reduce the dimensions by extracting the *r* leading time‐delay‐coordinates. Specifically, in the context of a delay‐embedded matrix *A*, the SVD can be expressed as *A* = *USV*′, where *U*, *S*, and *V*′ represent the orthogonal matrices that capture the dominant directions of variability in the rows and columns of *A*, and *S* denotes the diagonal matrix of singular values. Since *U* and *V*′ are unitary, their columns form a set of orthonormal vectors, which can be regarded as basis vectors. The columns of *V* are called the right singular vectors of *A* and provide the time series associated with each SVD component(Figure 1c). Taken's embedding theorem allows us to reconstruct a *k* −dimensional attractor, diffeomorphic to the original attractor with box‐counting dimension *d*_*a*_ (*k* > *d*_*a*_) from a single measurement. Therefore, in theory, a single measurement can uncover key features of the full dynamics of a complex biological system, which may reflect the dynamic trajectory underpinning variable seizure risk. Figure 1d demonstrates that the first three SVD coordinates reconstruct an attractor topology out of a single IEA time series for a sample patient.

The dynamics of a chaotic attractor can be decomposed into a linear model in the *r* − 1 leading SVD coordinates forced by low‐energy *r* coordinate (*V*_*r*_).^[^ ^23^ ^]^ An increase in forcing precedes switching and bursting phenomena in several analytic and real‐world systems. Accordingly, we hypothesize that seizures would reside in regions of the attractor with high non‐linearity, preceded by increased forcing in the reconstructed system. We used the line‐length measure (72‐h window) of *V*_*r*_ to quantify the gradual increase in the forcing's amplitude envelope. Highlights in Figure 1d show the regions with high forcing (normalized *V*_*r*_'s line‐length >0). In this example, we selected *r* = 4 as the forcing for this 3D system.

We use the device‐labeled ‘long events’ (LEs) as a proxy measurement of ictal activity since these are tuned by the clinicians to identify patient‐typical seizures and often represent electrographic seizures and correlate in frequency with seizure clinical frequency.^[^ ^25^ ^]^ The overlap between the patient‐labeled seizures and the long event shows the close relationship between the two measures in Figure 1a. Figure 1e shows in the sample patient that the long events cluster over a part of the manifold preceded by increased forcing.

**Figure 2:**
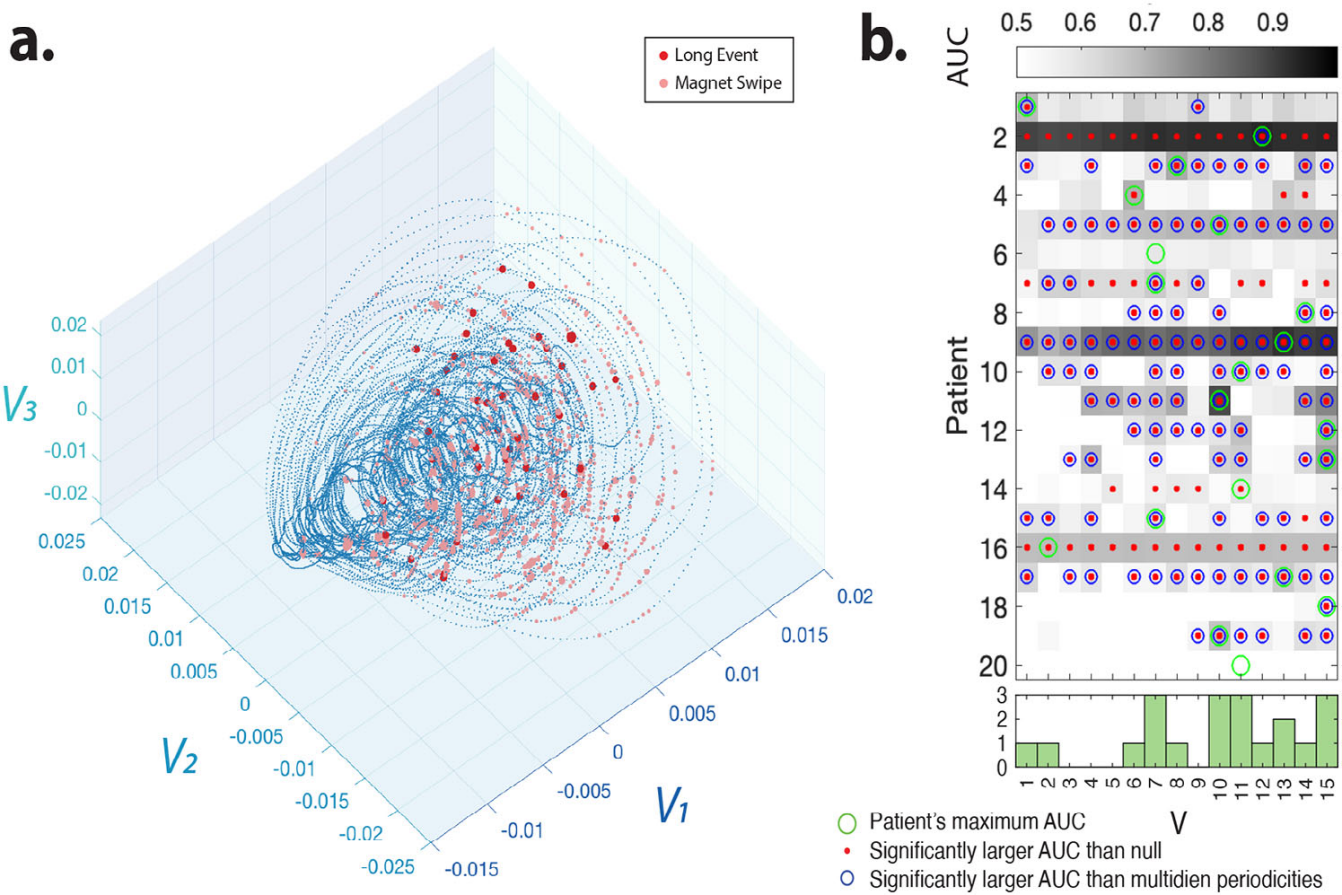
Delay‐embedded IEA‐count time series coordinates detect the seizure risk. a) Patient‐labeled seizures (red dots) and device‐labeled automatically detected 'long events' (LEs, pink dots) overlap with the region of the manifold marked by the increased forcing in Figure 1e. b) The mean daily area under the receiver operating characteristic curve (AUC) for detection of seizure risk (LEs) using different delay‐embedding coordinates over 50 repetitions of the analysis (see Experimental Section for classification details). The histogram (bottom panel) shows the number of patients with a peak AUC for a given SVD component. Coordinates derived from single SVD components predict LEs better than patient‐specific multi‐dien IEA cycles (see methods, peak paired *t*‐test between the SVD component providing max. AUC (green circles), and two cycles; detection mean AUC (std) for SVD = 0.67 (0.13), for slow cycles = 0.56 (0.14), p=9.8e‐04). Blue circles highlight each SVD that provides significantly better AUCs compared to multi‐dien cycle features (two‐sample *t*‐test, *p* < 0.05, Bonferroni corrected for multiple comparisons). Red dots show the mean AUC values that are significantly higher than those of the random null detection (two‐sample *t*‐test, *p* < 0.05, Bonferroni corrected for multiple comparisons across patients and coordinates. See Statistics section for more details on the null and permutation test).

Next, we trained a classifier to detect LEs for every patient using six months of delay‐embedded coordinates to test if the position in the phase space is predictive of seizure‐like electrocorticography patterns (see Experimental Section for details on the classification scheme). Our results show that in most patients, the higher frequency delay‐embedded coordinates are predictive of LE occurrence (permutation test *n* = 100 *p* < 0.05, see Statistics and Reproducibility for details on the random null model, and the Experimental Section for details regarding the classification scheme) (**Figure** **2**; Figure S2, Supporting Information)

It has been demonstrated that seizures tend to occur during the ascending phase of multi‐day cycles^[^ ^16^ ^]^ and that the phase of multi‐day cycles predicts seizure risk.^[^ ^18^ ^]^ Our results demonstrate that for most patients, delay‐embedded coordinates provide significantly more accurate detection of long events (Figure 2a,b.) than phase and amplitude of the identified main two slow multi‐day cycles (See Figure S1, Supporting Information and Experimental Section for more details on identifying the multi‐day cycles' peaks). Interestingly, *V*_1_, which corresponds to the coordinate with the slowest oscillations, only predicts the LEs of a few patients. A subset of patients reliably self‐reported seizures triggered externally by a magnetic wand. For these patients, delay‐embedded coordinates also allow the detection of self‐reported seizures, although different coordinates provide optimal detection across patients (Figure S3, Supporting Information).

**Figure 3:**
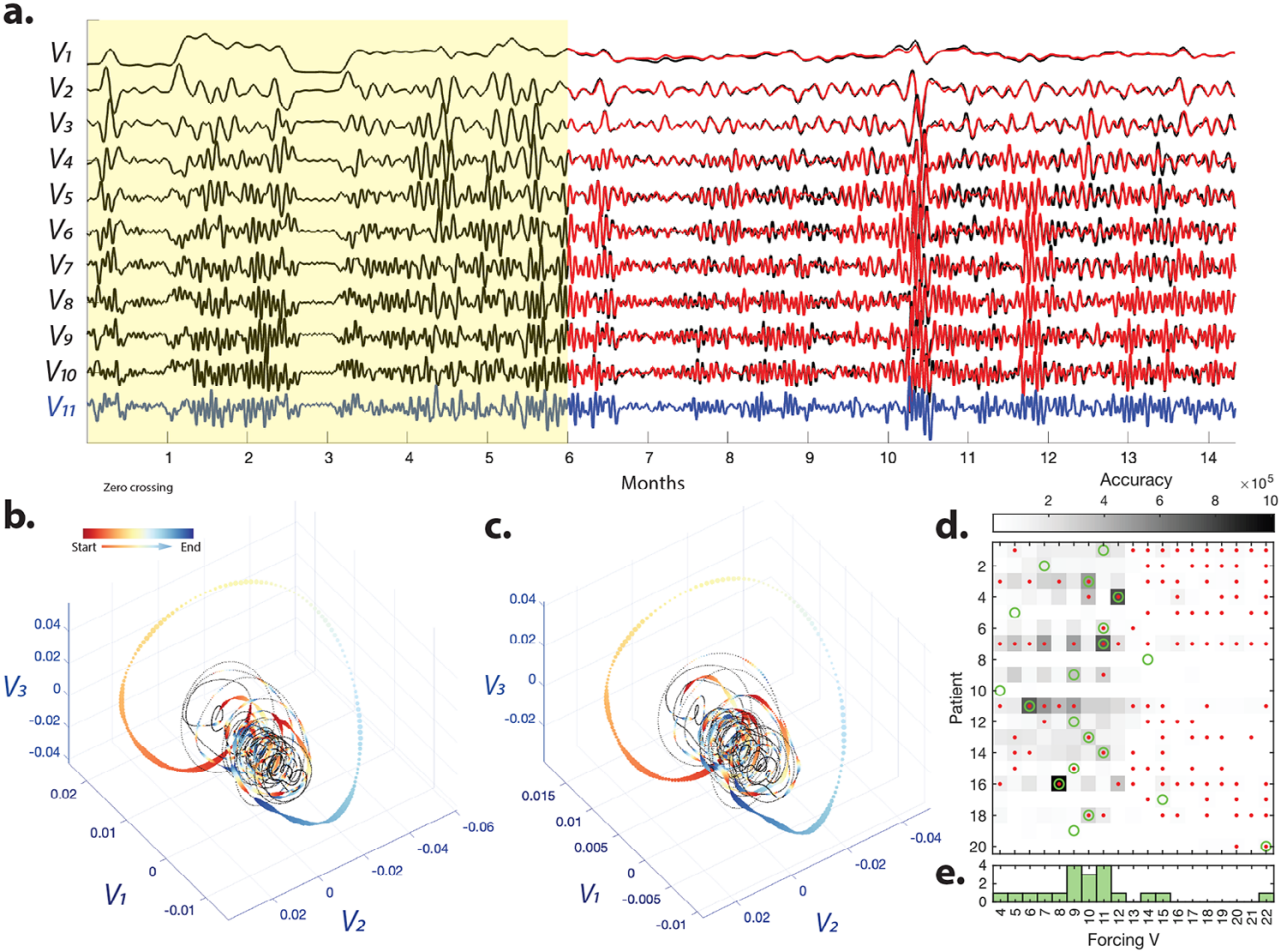
A linear model of the delay‐embedded coordinates driven by higher frequency forcing (i.e., *V*_*r*_) accurately predicts the future trajectory of multidien IEA‐periodicities. a) Black line shows the *V*_1_ to *V*_11_ of a sample patient. We trained a *r* − 1 = 10 D linear model using *V*_1_ to *V*_10_ and *V*_11_ (Blue line) as the forcing over a six‐month window (see Experimental Section for per‐processing details). The red lines show the predicted time series generated from the trained linear dynamical system driven by the known forcing (*V*_11_) over the test window (i.e., after the six‐month training window). The reconstructed embedded (b) and predicted attractors (c) from the test period. Instances of zero‐crossing in the normalized line length of the forcing are color‐coded. (d) Forecasting based on single SVDs performed better than forecasting based on multi‐dien cycle features (peak AUC for single SVD forecasts versus multi‐day cycle features differ in the 1‐day forecast: for single SVD, mean peak AUC (std) = 0.65 (0.08), for multi‐dien periodicities AUC (std) = 0.55 (0.1), paired *t*‐test *p* =8.3698e‐04; in the 7‐day forecast: single SVD mean peak AUC (std) = 0.61 (0.07), for multi‐dien periodicities mean AUC (std) AUC = 0.55 (0.08, paired), paired *t*‐test *p* =1.3280e‐04). The plot shows the accuracy (the inverse of mean squared error) of the predicted leading coordinate (*V*_1_) time series for systems of different sizes with *V*_4_ to *V*_22_ as forcing. Red dots indicate significantly higher accuracy than those of the future trajectory predictions of the phase‐randomized IEA‐count null time series (*p* < 0.05, two‐tailed, *n* = 50 iterations, FDR corrected for multiple comparisons across patients and delay‐embedded coordinates). Green circles highlight the maximum accuracy for each subject. The histogram (bottom panel) indicates the number of subjects with maximum accuracy for each forcing.

Within the HAVOK framework, the low‐energy forcing drives the leading delay coordinates. This implies that the forcing can be used to causally drive the dynamics and enable forecasting the trajectory of the full system. To test this hypothesis, we apply the HAVOK framework to train a linear model based on a six‐month window. Next, we simulate the fitted linear system dynamics using the known future forcing time series (i.e., *V*_*r*_). As seen in **Figure** **3** in a sample patient, the simulated *V*_1_ to *V*_10_ time series using the *V*_11_ as the forcing closely match the delay embedded coordinates and reconstructs the embedded attractor. As seen in Figure S4 (Supporting Information, this forcing (*V*_11_) displays fat‐tailed distributions corresponding to intermittent and rare events.

This close fit suggests that an intermittently forced linear model can accurately capture the chaotic dynamics underpinning ictogenicity and highlight the identified forcing's potential for seizure risk forecast. Moreover, we quantified the accuracy – measured as the inverse of the mean squared error – of predicted trajectories of the leading coordinate (*V*_1_) in Figure 3d and the three leading delay‐embedded coordinates of the IEA‐count time series for all patients in Figure S5 (Supporting Information). As seen in Figure 3d, the accuracy of the predicted leading coordinate (*V*_1_) increases for higher dimensional linear systems, followed by a sharp drop in accuracy at the 12‐D system (*V*_13_ as forcing) for all patients. Figure S6 (Supporting Information) demonstrates that *U*_10_ to *U*_12_ have prominent 25‐hour cycle peaks for almost all patients and *U*_13_ corresponds to faster cycles (period < 25 h).

The delay‐embedded coordinates and the forcing time series are extracted from the entire IEA‐count time series using the singular value decomposition of the delay‐embedded Hanekl matrix. Alternatively, we can extract the forcing time series of the test time series directly using the basis vector of the *U* matrix estimated from the six‐month window. Specifically, it is possible to measure forcing (*V*_*r*_) from a streaming time series by convolution with the *r*_*th*_ mode (column *r* of matrix *U*). Our results show that similarly, the convolution‐based estimated forcing accurately predicts trajectories of the three leading delay‐embedded coordinates of the IEA‐count time series (see Figure S7, Supporting Information for details). Like the above‐mentioned results, the forcing corresponding to the daily cycles provides the most accurate predictions. Together, this close fit suggests that an intermittently forced linear regression model can accurately capture the chaotic dynamics underpinning ictogenicity and highlights the estimated forcing's potential for seizure risk forecast.

**Figure 4:**
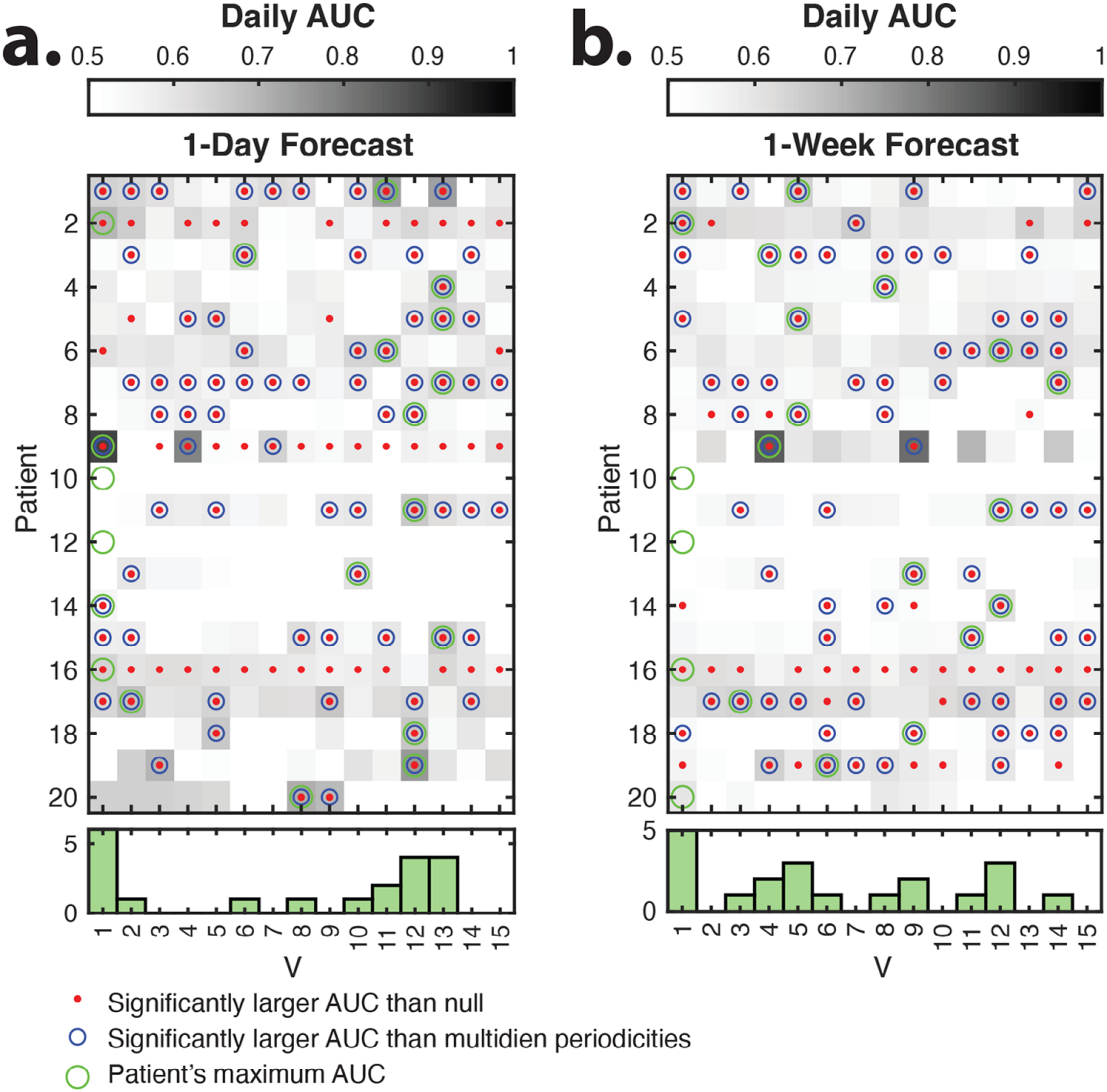
Delay‐embedded coordinates of the IEA‐count time series enable forecast of the seizure risk. The mean daily Area Under the Receiver Operating Characteristic Curve (AUC) for one day (a) and one week (b) forecast of seizure risk (long events) using different delay‐embedding coordinates over 50 repetitions of the analysis (see Experimental Section for classification details). Red dots show the mean AUC values that are significantly higher than those of the random null forecast (two‐sample *t*‐test, *p* < 0.05, Bonferroni corrected for multiple comparisons across patients and coordinates. See Statistics section for more details on the null and permutation test). Blue circles show the mean AUC values that are significantly (two‐sample *t*‐test, *p* < 0.05, Bonferroni corrected for multiple comparisons) higher than the AUC values calculated from the two slow peak features (i.e., their amplitude and phase). Green circles show the maximum mean AUC across all coordinates for each patient. Histograms (bottom panels) indicate the number of subjects with maximum AUC for each forcing.

Finally, to test the hypothesis that the delay‐embedded coordinates enable the forecast of future seizure risk, we train a classifier using the time‐shifted long event time series (1‐ to 7‐day shifts) and measure the forecast accuracy. In most patients, delay‐embedded coordinates forecast LEs daily risk in the next day to week significantly better than randomized nulls (*t*‐test, *p* < 0.05, Bonferroni corrected for multiple comparisons across patients, **Figure** **4**). For the majority of patients, the highest AUCs for one‐day forecast were achieved for faster delay‐embedded coordinates (*V*_*r*_), *r* > 6 period length < = 40*h* 4a). Overall, this method performed better than forecasting based on the main two identified multi‐day four). Similar results are observed for longer 2‐ to 7‐day forecasts of long events (Figure S8, Supporting Information) and patient‐labeled seizures (Figure S9, Supporting Information). Together, these results validate the utility of the identified delay coordinates and the higher frequency forcing for improving the accuracy of the seizure risk forecast.

## 3 Discussion

In this study, we have introduced a novel data‐driven model‐based approach to approximate the dynamics of a putative dynamical system driving slow multi‐day rhythms in seizure risk in individual patients undergoing long‐term electrocorticography monitoring for seizures. We aimed to reconstruct a linear dynamical system intermittently influenced by higher‐frequency low‐energy drivers based on the complex long‐term IEA‐count time series obtained from the RNS device. Our findings demonstrate the effectiveness of this framework in capturing the causal relationship between the higher frequency forcings and the slow multi‐day oscillations and accurately predicting the system's future trajectory based on the modeled dynamics. By leveraging the high‐frequency delay‐embedded coordinates as drivers, we successfully drive the slow multi‐day oscillations in silico and accurately reconstruct the future trajectory of the full system in most patients, shedding light on the complex interactions underlying seizure propensity dynamics.

Our study presents a novel framework for understanding the slow oscillations observed in epilepsy by linking them to dynamics evolving at multiple timescales. This causal relationship, identified through our HAVOK framework, provides a new perspective on seizure dynamics and allows for the development of dynamic models from individual patient data to characterize seizure occurrence based on the complex set of dynamics observed in ultra‐long‐term data. Specifically, our model identifies the “forcing” signal as a critical feature driving sudden changes in daily seizure risk, shifting the current focus of the field from predicting the phase of slow oscillations to examining these abrupt transitions in the ongoing dynamics of the system. This approach expands the search for possible measurable correlates of impending transitions toward the high seizure probability state since these may occur at the level of comparatively fast oscillatory signals, which are filtered out by currently established approaches.

Importantly, our results bear clinical significance as they showcase the potential of our approach to enhance the detection and forecasting of impending seizures over extended periods with patient‐specific data‐driven models. By leveraging the insights gained from our model, we demonstrate improved capabilities in identifying and anticipating seizure events over several days, providing valuable information for clinicians and patients. This work establishes a framework for quantitatively characterizing relationships between circadian and slow multi‐day rhythms, seizure risk dynamics, and external drivers. This holds promise for developing more reliable seizure risk forecasting models and personalized interventions based on patient‐specific physiological recordings.

Forecasting epileptic seizure risk dynamics poses a formidable challenge for traditional modeling approaches that either oversimplify or obscure critical transitions. Classical linearization techniques, such as Taylor series expansions^[^ ^26^ ^]^ and Dynamic Mode Decomposition (DMD),^[^ ^27^ ^]^ are constrained by locality or an inability to resolve intermittent transitions. At the same time, data‐driven machine learning models trade interpretability for predictive power. HAVOK circumvents these limitations by constructing a globally valid linear representation of nonlinear dynamics through delay‐embedding, avoiding the restrictive equilibria of Taylor approximations and DMD's omission of forcing terms. Unlike sparse identification of nonlinear dynamics,^[^ ^20^ ^]^ which requires predefined nonlinear features or black‐box models^[^ ^28^ ^]^ such as Long short‐term memory (LSTM) recurrent neural networks^[^ ^29^ ^]^ that mask mechanisms, HAVOK achieves comparable forecasting accuracy without prior assumptions while explicitly linking intermittent forcings–such as circadian drivers–to transitions into seizure‐prone states. By unifying global linearization, interpretable dynamics, and predictive robustness, HAVOK offers a transformative framework for identifying the nonlinear attractor topology underlying seizure risk, adding to the tools now available for improving clinical prediction.

Our work highlights the advantages of adopting a linear model to capture the interactions between slower modes driven by faster modes and the low‐energy external forcing compared to the conventional wavelet‐based analysis of the IEA‐count time series. This novel approach offers several key benefits. First, it provides a data‐driven and model‐based improvement on established methods, currently based on the subjective selection of peaks in the IEA wavelet power spectra (e.g.,^[^ ^16^, ^30^ ^]^), which often relies on arbitrary criteria to identify the slow oscillatory modes. Second, unlike our model‐based framework, wavelet analysis fails to unveil the intricate relationship between the slower and faster dynamics. In contrast, our proposed linear model effectively uncovers this crucial connection, shedding light on the underlying mechanisms driving the observed phenomena. Figure S1 (Supporting Information) illustrates how spectral analysis of the IEA‐count time series wavelet decomposition can provide additional insight into the frequency of amplitude modulations at various scales. However, these wavelet analysis results remain purely descriptive, lacking our linear model's explanatory power and predictive capabilities.

Previous studies have highlighted the clinical significance of IEA cycles, specifically the correlation between the phase of multidien periodicities and seizure risk.^[^ ^14^, ^16^ ^]^ However, these proof‐of‐concept studies primarily rely on non‐causal filtering of the IEA‐count time series (e.g.,^[^ ^18^ ^]^), limiting their practical application for real‐time seizure risk forecasting. The critical advantage of our findings and framework lies in its ability to directly predict the emergence of slow oscillations from their higher‐frequency drivers, such as daily cycles, using the modeled system. This approach eliminates the lengthy delays introduced by causal low‐pass filtering when forecasting the impending increase in IEA counts. Instead, our method leverages faster oscillations, which theoretically exhibit shorter delays, resulting in more timely and accurate seizure risk predictions.

The model‐based approach presented here builds on and is complementary to previously published approaches that aim to forecast seizures from ultra‐long‐term EEG recordings.^[^ ^28^, ^31^ ^]^ A large crowd‐sourced seizure prediction competition achieved test set seizure prediction based on pre‐ictal data with AUC values of >0.80 based on multichannel intracranial continuous ECoG recordings from the Neurovista device, with different machine learning algorithms performing best for individual patients.^[^ ^28^ ^]^ Forecasting of seizures based on multidien cycles of interictal activity data from Neuropace devices has been reported to achieve an AUC of up to 0.70.^[^ ^18^ ^]^ In our dataset, this multidien forecasting is outperformed by the HAVOK‐based approach for 15 (1‐day forecasts) to 16 (1‐week forecast) of 20 patients (Figure 4). Note that our comparison focused specifically on the phase and amplitude of slow multidien cycles relative to the estimated forcing using HAVOK, with the primary aim of demonstrating the benefit of modeling the relationship between fast and slow parameters. We show how the circadian cycles in several patients drive the slow oscillations, which might contribute to an extended machine learning model incorporating a comprehensive range of slow to ultra‐fast rhythms as seen in Ref. [18], achieving similarly high predictive power. Additionally, our model‐based approach integrates observations of cyclical statistical regularities that lead to the description of multiday cycles into a dynamical systems framework. Such frameworks may be necessary to integrate multimodal information, such as, e.g., complementary physiological measures.^[^ ^13^, ^32^ ^]^ into a single, interpretable predictive model. Importantly, from such models, fast‐evolving features that predict transitions between dynamical regimes may be identifiable in the future and may help translate these candidate measures for seizure prediction into clinically useful tools.

Our work identifies key changes in dynamics evolving at faster time scales as predictors that can be used in the forecasting of changes in slow transitions. Identifying faster markers of impending transitions in the slow‐evolving brain state would offer a critical window for potential early intervention in high‐risk seizure states. These faster fluctuations identified from RNS data evolve at similar time scales to physiological systems that significantly affect neuronal circuit physiology, such as sleep‐wake cycles,^[^ ^33^ ^]^ and hypothalamic‐pituitary‐adrenal axis function.^[^ ^13^ ^]^ However, whether these relate to the specific changes observed as “forcing” elements in our analysis is currently unclear and subject to future research.

Posing the multi‐scale IEA cycles as outputs of intermittently forced attractors presents a novel avenue for comprehending the drivers of ictogenicity. By examining the estimated slow coordinates and forcing, we can uncover connections to physiological factors (e.g., metabolic or hormonal rhythms^[^ ^34^ ^]^) or pathophysiological processes. Furthermore, our model‐based approach provides a framework for testing hypotheses regarding external or internal factors. For instance, we can model and predict the impact of external interventions, such as drugs or neurostimulation treatments, on the impulse response of the modeled system and IEA cycles. Similarly, future research could aim to enhance prediction accuracy by explicitly adding known external stressors, such as weekly stressors, to the model.

The approach here provides a foundational framework to capture complex dynamic changes in statistical features of the intracranial data. The relationship of these changes to a fuller description of ongoing cerebral activity ‐ such as the full EEG spectrum ‐ is yet to be explored. Similarly, The relationship to hormonal,^[^ ^35^ ^]^ metabolic,^[^ ^36^ ^]^ or other physiological changes^[^ ^37^ ^]^ – all of which have been shown to affect whole‐brain dynamics and occur at similar time scales – and their potential clinical implications remain key questions for future research.

Interestingly, our findings indicate that changes in the circadian IEA cycles and the high‐dimensional model of the IEA‐count time series offer the most accurate forecast of future IEA‐count trajectories for most patients. However, most patients' circadian IEA cycles were not the most predictive of future seizure risk. Instead, other slower oscillations demonstrated better accuracy in forecasting seizure risk in several patients. These results suggest that while circadian forcing can effectively predict the future of slow oscillations, the clinical manifestation of seizures may be better explained and mechanistically linked to a few or multiple slower coordinates within the system.

Whilst overall, our approach shows predictive validity for a majority of patients, there is clear heterogeneity in the patients, the individual shape of the captured attractors, and the time scales most informative for the predictive tasks. Brain activity is significantly shaped by different physiological processes that may play a role in interacting with these observed temporal features ‐ ranging from sleep‐wake cycles^[^ ^33^ ^]^ and diurnal variation of cortisol levels affecting daily fluctuations,^[^ ^13^ ^]^ to glucose variability,^[^ ^36^ ^]^ slow changes in cytokine release (Vezzani et al., 2011), and hormonal changes^[^ ^35^ ^]^ affecting slower variability. A detailed characterization of all these factors over long time scales in humans has yet to be achieved. In this context, HAVOK provides a powerful tool for identifying and reconstructing attractor dynamics. In this case, the identification of key dynamical changes that evolve over fast time scales identifies high amplitude fluctuations in daily physiological measures as a key potential target for future biomarker research.

## 4 Limitations

Long‐term sampling of intracranial activity with implantable devices offers a unique insight into the long‐term trajectories of epileptiform brain activity in individual patients. However, these devices are currently only available to a small set of patients, limited to those where the device is clinically indicated and who have access to it through their local healthcare system. This limits the cohort size to typically 10‐40 patients,^[^ ^16^, ^38^, ^39^, ^40^, ^41^ ^]^ including in the study presented here. Because of the extensive within‐patient sampling, this small cohort of patients still provides access to a large volume of data (sampling from at least > 8000 hours of recording time for each patient) but generalisability to other patient groups – particularly to patients living with epilepsy syndromes that are not included in the cohort – may be limited. Therefore, the aim of long‐term individual sampling is focused on mapping the trajectories of the dynamics of individual patients' brains and developing tools that facilitate this. Our current findings are most relevant to patients with implantable devices capable of long‐term ECoG monitoring. To take advantage of this unique opportunity, our study focused on reconstructing individual patient attractors and predicting their specific seizure risk trajectories. While this approach offers a high degree of personalization, it's important to note that other readily available physiological measures, such as heart rate variability, have shown promise in capturing cyclical variations in seizure risk. Incorporating these additional factors into future models could further enhance individualized seizure risk prediction. Additionally, future approaches that integrate a more comprehensive set of physiological signals (e.g., external stressors or hormonal cycles as external inputs to the model) may allow models to extend beyond the specific cohort investigated here and ultimately make similar approaches more generalizable across a broader patient population. In this study, we do not consider the impact of electrical stimulations delivered by the responsive neurostimulation devices on the IEA‐count cycles. Recent studies have shown that neuromodulation can alter cycles,^[^ ^42^ ^]^ and stimulation can suppress EEG signal features.^[^ ^43^, ^44^ ^]^ Therefore, instead of a fixed attractor, the IEA time series may be better modeled as following an attractor trajectory with slowly changing underlying parameters. Distinguishing the “faster” multiday fluctuations along the attractor from the “slower” changes in trajectory shape will require significantly more data (e.g., device parameters, stimulation records, and clinical history) than available for the analysis presented here. Nevertheless, our framework offers a model‐based avenue for future work to explore the interplay between latent pathophysiological variables and electroceutical interventions. There are other important caveats to consider regarding our findings, such as the unaccounted changes in the detection and stimulation parameters of the RNS device, as well as the use of long events and patient‐labeled seizures as a proxy for clinical seizures, which have previously been shown to correlate with clinical seizure frequency.^[^ ^25^ ^]^ Clinically, RNS device electrodes are typically implanted to target the presumed seizure onset zones associated with specific seizure types, which are assumed to remain stable throughout the recording period. However, if significant changes occur in the spatial distribution of seizure onset, the implanted electrodes may not capture these shifts. This limitation could adversely affect the quality of IEA sampling, compromise the accuracy of the computational model, and reduce the therapeutic effectiveness of the device. Additionally, the small cohort sample size may limit generalizability. Nonetheless, our study contributes to the expanding knowledge base on slow multi‐day rhythms in epilepsy, providing evidence for the clinical significance of accurately modeling and predicting seizure dynamics. The implications of our findings extend beyond theoretical implications, holding promise for advancements in seizure management and improving patient care.

## 5 Experimental Section

### Patient Population Information

Retrospective analysis was conducted on longitudinal hourly counts of detected interictal epileptiform activity (IEA) based on long‐term electrocorticography recordings obtained from a subset of 20 out of 28 patients with drug‐resistant epilepsy who underwent implantation of the RNS System (NeuroPace, Inc., Mountain View, CA) at the Hospital at the University of Pennsylvania. The inclusion criteria required patients to had a minimum of one year of IEA recordings, resulting in the exclusion of eight patients. The 20 patients included in this study were implanted with the RNS device between August 2015 and January 2022. Before participating in this study, all patients provided written informed consent following the Institutional Review Board of the University of Pennsylvania.

### RNS IEA‐Count Data Preprocessing

In addition to providing real‐time responsive neurostimulation, the RNS system keeps track of the hourly number of interictal epileptiform activity (IEA). To ensure the early and effective detection of seizures, the clinicians manually tune the detection parameters of the RNS device or patients' office visits. Consequently, the detected hourly IEA counts could vary notably pre and post‐office visits. To address this issue, (z‐score) the IEA‐count time series between each office visit was normalized. The first five months of data were also removed to account for implantation effects and other factors that contribute to high variability commonly observed early in the IEA‐count time series.

### Identifying Multi‐Day Cycles' Frequency Peaks

A methodology similar to that described in Baud et al. (2018) was employed to extract multi‐day cycles from the IEA hourly counts.^[^ ^16^ ^]^ In brief, for each patient, a wavelet transform was applied to the IEA‐count time series and identified the peaks in the average power across time scales (peaks in the periodogram) ranging from 3–45 days. Specifically, two multi‐day peaks were selected with the largest powers, one between 3–11 days and the slowest between 7–45 days.

### Hankel Alternative View of Koopman (HAVOK) Analysis

The Hankel alternative view of Koopman (HAVOK) methodology and analysis developed by Brunton et al.^[^ ^23^ ^]^ was employed. Specifically, a delay‐embedding (Hankel) matrix was created from the IEA‐count time series by stacking 100 delayed (each row one time point delayed compared to the previous row). The stacking parameter was tuned to capture the dominant frequencies in the time series. Namely, too small or too big stacking values could lead to loss of sensitivity to low or high‐frequency modes, respectively. Next, to find the eigen‐delay‐embedding coordinates, we applied Singular Value Decomposition (SVD) to the Hankel matrix.

Hankel matrices offer a powerful alternative to other approaches for inferring multi‐scale temporal structures in high‐dimensional, noisy data. Unlike alternative approaches such as mutual information^[^ ^45^ ^]^ and false nearest neighbors,^[^ ^46^ ^]^ here temporal dependencies were systematically captured across multiple time scales, effectively transforming time series data into a structured representation that reveals underlying dynamical patterns. Singular value decomposition then provided a mathematically rigorous means of decomposing this matrix, extracting principal components that capture the most significant dynamical modes, reducing noise, and revealing low‐dimensional structures that traditional methods might miss. This approach was particularly powerful in seizure prediction, where conventional analysis techniques could obscure subtle, complex temporal signatures. The HAVOK method leverages the Hankel matrix's structured representation to systematically embed time‐delayed signals, with a specific approach of delaying each row by one timepoint relative to the previous row. This strategy created a matrix that captures the system's temporal dynamics with precision aligned with the SVD decomposition. By constructing the matrix this way, HAVOK enabled the linearization of nonlinear dynamical systems, provided robust dimensionality reduction, and extracts low‐dimensional representations that preserve the system's fundamental dynamical characteristics. With adequate temporal depth, the basis vectors in U take on polynomial‐like forms, which aid in the accurate reconstruction of long‐term seizure risk.^[^ ^23^ ^]^ While the standard implementation suggests delaying one timepoint per row, alternative delay strategies were theoretically possible, though they would require rigorous validation to maintain the method's core reconstruction properties and ensure a meaningful representation of the underlying dynamical system.

The Hankel matrix, denoted as *A*, could be decomposed using Singular Value Decomposition (SVD) into the form *A* = *USV*′, where *U*, *S*, and *V*′ represent orthogonal matrices that capture the primary directions of variability in the rows and columns of *A*. The matrix *S* contains the singular values along its diagonal. Importantly, the columns of the unitary matrices *U* and *V*′ form sets of orthonormal vectors, which could be interpreted as basis vectors. Specifically, the columns of matrix *V* correspond to the right singular vectors of *A*, providing the time series associated with each SVD component.

Figure S10 (Supporting Information) shows the first five columns of *U*. Note that although, as expected, *U*_1_ to *U*_5_ captures the lowest to faster oscillations, circadian cycles are (24‐h cycles) present in all the basis vectors. This is due to the relatively high power of circadian cycles in the IEA‐count time series. As mentioned, the HAVOK framework aims to decompose the chaotic and non‐linear time series into a linear system forced by low‐energy, high‐frequency modes. However, the strong presence of high‐power circadian IEA‐count cycles poses an issue for this framework and could lead to the aforementioned mixing of frequencies in the basis vectors. Moreover, it had been observed that models are more accurate and predictive when the basis vector *U* resembles polynomials of increasing order, as shown in Figure 1d.

To address this issue, the IEA‐count time series was low‐pass (3 days) filtered using a non‐causal filter (FIR filter, Order = 100). Figure 1d. shows a sample patients' *U*_1_ to *U*_4_ of the low‐pass filtered time series, which showed these basis vectors now only represent the lowest frequencies. The main frequency peak of the *U* basis vectors identified using Fast Fourier transformation also reveals the monotonous decrease in the main frequency of *U*_*r*_ basis vectors for higher values of *r* (Figure S6, Supporting Information). These observations were the basis for choosing the low‐pass filtering reprocessing step.

Next a linear model was constructed for the first *r* − 1 variables of *V* driven by *V*_*r*_:

(1) $$V\left(t+1\right)=AV\left(t\right)+BV_{r}\left(t\right)$$

here *V*(*t*) = [*V*_1_, *V*_2_, …, *V*_*r* − 1_]^*T*^ is the *r* − 1 leading delay‐embedded coordinates time series at time point *t*, *V*_*r*_(*t*) act as the input or forcing, and *A* and *B* are the system and input matrices, respectively. The system and input parameters were estimated from a six‐month IEA‐count time series window. Specifically, cripts provided by Ref. [20] were used, which fit a discrete linear system by solving a least‐squares problem. Next, the full future trajectory of the modeled system (i.e., *V*(*t*)) was stimulated using the *V*_*r*_(*t*) time series after the six‐month point. The mean squared error (MSE) of the predicted V(t) was separately quantified for each dimension. Here, the inverse of the mean squared error (1/MSE) was reported to reflects model accuracy; i.e., low values in mean squared error reflect a highly accurate model. The accuracy of the predicted first leading three dimensions w, *V*_1_, *V*_2_, and *V*_3_, was reported in Figure S5 (Supporting Information). The convolution of the basis vector *U*_*r*_ estimated from the first six months with the full IEA‐count time series was also explored to extract the forcing (*V*_*r*_) after the six‐month time points. Figure S7 (Supporting Information) shows the accuracy of the predicted leading system dimensions using the convolution‐based estimated forcing.

### Seizure Risk Detection and Forecast

Two identified slow multi‐day peaks were examined to investigate the utility of amplitude and phase features in detecting and forecasting seizure risk. For the detection analysis, a non‐causal filter (Finite Impulse Response (FIR) filter, order = 100) was applied to the preprocessed IEA‐count time series centered at the slow multi‐day peak frequency ± one day. However, for the forecast analysis, a causal filter was leveraged and shifted the long IEA events (or patient‐labeled seizures) by *x* days (ranging from *x* = 1 to 7) to assess the predictive power of features in real‐time scenarios. The instantaneous phase of the filtered time series was then calculated using the Hilbert transform.

Additionally, The performance of delay‐embedded coordinates and forcing in seizure detection and forecasting was explored. In the detection analysis, the delay‐embedded coordinates time series *V*_1_, …, *V*_*r*_ and their instantaneous phase as features was used to predict seizure risk. In the forecast analysis, apart from shifting the seizure labels, the forcing time series *V*_*r*_ were directly extracted from the IEA‐count time series using convolution with *U*_*r*_. The convolution‐derived *V*_*r*_ time series and its instantaneous phase as features were exclusively utilized.

Slow multi‐day and delay‐embedded coordinates features were used separately to train a regression model using a bagging ensemble of decision trees (minimum leaf size = 8) and a support vector machine (SVM) regressor. After removing an initial five‐month data window (to account for early implant effects), the model was trained by using the first six months of patients' recording. The model's accuracy was tested by using the Area under the Receiver operating characteristic Curve (AUC) on the remaining time series. In addition to hourly results, the daily accuracy was also examined by training the model using the average feature values and the total sum of long IEA event counts (or patient‐labeled seizures) over 24 periods.

### Statistics

The statistical significance of the detection and forecast AUC values against the null model results were tested. Specifically, the long IEA‐count time series were randomly shuffled to train and test the accuracy of the above‐mentioned model. Distributions of empirical and null AUC values were created by repeating the analysis 50 times. *t*‐test (*p* < 0.05, two‐tailed, FDR corrected for multiple comparisons across patients and delay‐embedded coordinates) was used to assess whether the AUC values were significantly larger than the null model's. Similarly, the performance of the slow multi‐day features against the delay‐embedded features were compared by creating two distributions of AUC values (*n* = 50 iterations) and the subsequent *t*‐test (*p* < 0.05, two‐tailed, FDR corrected for multiple comparisons across patients and delay‐embedded coordinates).

Statistical testing was employed to determine the significance of the accuracy in projected trajectories of the modeled linear system, as described in the “Hankel alternative view of Koopman (HAVOK) analysis” section of the Materials and Methods. This testing allowed us to establish whether the predicted trajectories performed significantly better than what would be expected by chance alone. Specifically, the linear model on the phase‐randomized IEA‐count time series was repeatedly (*n* = 50 iterations) trained and tested to create a distribution of null accuracy results. As mentioned above, the prediction accuracy of leading delay‐embedded coordinates *V*_1_, *V*_2_, and *V*_3_ separately was calculated by using the inverse mean squared error. Finally, *t*‐test (*p* < 0.05, two‐tailed, FDR corrected for multiple comparisons across patients and delay‐embedded coordinates) was used to identify significantly high accuracy projections.

## Conflict of Interest

The authors declare no conflict of interest.

## Author Contributions

R.E.R., B.S., B.L., and A.A. contributed to the conceptualization of this study. R.E.R. was involved in methodology and writing. B.S. handled data preprocessing, methodology, data analysis, and writing. B.L. contributed to writing. A.A. participated in methodology, data analysis, and writing.

## Supporting information

Supporting Information

## Data Availability

The data that support the findings of this study are available on request from the corresponding author. The data are not publicly available due to privacy or ethical restrictions.

## References

[1] C. J. Doherty , S. A. Kay , Annu. Rev. Genet. 2010, 44, 419. 20809800 10.1146/annurev-genet-102209-163432 PMC4251774

[2] S. Panda , M. P. Antoch , B. H. Miller , A. I. Su , A. B. Schook , M. Straume , P. G. Schultz , S. A. Kay , J. S. Takahashi , J. B. Hogenesch , Cell 2002, 109, 307. 12015981 10.1016/s0092-8674(02)00722-5

[3] J. Siegelová , G. Cornélissen , A. Havelková , J. DUSEK , P. Vank , P. DOBSAK , Noninvasive Methods of Cardiology, Masaryk University, Brno, Czech Republic 2014, pp. 109–24.

[4] F. C. Baker , H. S. Driver , Sleep Med. 2007, 8, 613. 17383933 10.1016/j.sleep.2006.09.011

[5] Y. Touitou , E. Haus , Biologic rhythms in clinical and laboratory medicine, Springer Science & Business Media, Berlin, Heilberg 2012.

[6] M. Gallerani , M. Pala , U. Fedeli , Heart Fail. Clin. 2017, 13, 703. 28865780 10.1016/j.hfc.2017.05.007

[7] C. L. Ehlers , E. Frank , D. J. Kupfer , Arch. Gen. Psychiatry 1988, 45, 948. 3048226 10.1001/archpsyc.1988.01800340076012

[8] T. A. Wehr , Transl. Psychiatry 2018, 8, 151. 30104658 10.1038/s41398-018-0203-x PMC6089884

[9] M. A. Riva , L. Tremolizzo , M. Spicci , C. Ferrarese , G. De Vito , G. C. Cesana , V. A. Sironi , J. Hist. Neurosci. 2011, 20, 65. 21253941 10.1080/0964704X.2010.481101

[10] P. J. Karoly , R. E. Stirling , D. R. Freestone , E. S. Nurse , M. I. Maturana , A. J. Halliday , A. Neal , N. M. Gregg , B. H. Brinkmann , M. P. Richardson , A. La Gerche , D. B. Grayden , W. D. Souyza , M. J. Cook , EBioMedicine 2021, 72. 10.1016/j.ebiom.2021.103619 PMC8517288 34649079

[11] M. Morrell , Neurology 2011, 77, 1295. 21917777 10.1212/WNL.0b013e3182302056

[12] R. Bajorat , M. Wilde , T. Sellmann , T. Kirschstein , R. Köhling , Epilepsia 2011, 52, e118. 21801169 10.1111/j.1528-1167.2011.03200.x

[13] P. J. Karoly , V. R. Rao , N. M. Gregg , G. A. Worrell , C. Bernard , M. J. Cook , M. O. Baud , Nat. Rev. Neurol. 2021, 17, 267. 33723459 10.1038/s41582-021-00464-1

[14] M. G. Leguia , R. G. Andrzejak , C. Rummel , J. M. Fan , E. A. Mirro , T. K. Tcheng , V. R. Rao , M. O. Baud , JAMA Neurol. 2021, 78, 454. 33555292 10.1001/jamaneurol.2020.5370 PMC7871210

[15] V. R. Rao , M. G Leguia , T. K. Tcheng , M. O. Baud , Epilepsia 2021, 62, S15. 32738157 10.1111/epi.16611

[16] M. Baud , J. K. Kleen , E. A. Mirro , J. C. Andrechak , D. King‐Stephens , E. F. Chang , V. Rao , Nat. Commun. 2018, 9, 1. 29311566 10.1038/s41467-017-02577-y PMC5758806

[17] I. Robertson‐Dixon , M. J. Murphy , S. G. Crewther , N. Riddell , Life 2023, 13, 10. 10.3390/life13101968 PMC10608196 37895351

[18] T. Proix , W. Truccolo , M. G. Leguia , T. K. Tcheng , D. King‐Stephens , V. R. Rao , M. O. Baud , Lancet Neurol. 2021, 20, 127. 33341149 10.1016/S1474-4422(20)30396-3 PMC7968722

[19] K. J. Friston , A. D. Redish , J. A. Gordon , Comput. Psychiatry (Cambridge, Mass.) 2017, 1, 2. 10.1162/CPSY_a_00001 PMC5774181 29400354

[20] S. L. Brunton , J. L. Proctor , J. N. Kutz , Proc. Natl. Acad. Sci. 2016, 113, 3932. 27035946 10.1073/pnas.1517384113 PMC4839439

[21] S. H. Rudy , S. L. Brunton , J. L. Proctor , J. N. Kutz , Sci. Adv. 2017, 3, e1602614. 28508044 10.1126/sciadv.1602614 PMC5406137

[22] M. Scheffer , J. Bascompte , W. A. Brock , V. Brovkin , S. R. Carpenter , V. Dakos , H. Held , E. H. van Nes , M. Rietkerk , G. Sugihara , Nature 2009, 461, 53. 19727193 10.1038/nature08227

[23] S. L. Brunton , B. W. Brunton , J. L. Proctor , E. Kaiser , J. N. Kutz , Nat. Commun. 2017, 8, 19. 28559566 10.1038/s41467-017-00030-8 PMC5449398

[24] F. Taken , in Dynamical Systems and Turbulence, Warwick 1980, (Eds.: D. A. Rand , L. S. Young ), Springer, Berlin, Heidelberg 1981, pp. 366–381.

[25] I. H. Quraishi , M. R. Mercier , T. L. Skarpaas , L. J. Hirsch , Epilepsia 2020, 61, 138. 31849045 10.1111/epi.16412 PMC7003822

[26] S. H. Strogatz , Nonlinear dynamics and chaos: With applications to physics, biology, chemistry, and engineering, second edition, 2 edition, CRC Press, London, England 2019.

[27] J. N. Kutz , S. L. Brunton , B. W. Brunton , J. L. Proctor , Dynamic mode decomposition: Data‐driven modeling of complex systems, Society for Industrial & Applied Mathematics, New York, NY 2016.

[28] L. Kuhlmann , P. Karoly , D. R. Freestone , B. H. Brinkmann , A. Temko , A. Barachant , F. Li , G. Titericz , Jr, B. W. Lang , D. Lavery , K. Roman , D. Broadhead , S. Dobson , G. Jones , Q. Tang , I. Ivanenko , O. Panichev , T. Proix , M. Náhlík , D. B. Grunberg , C. Reuben , G. Worrell , B. Litt , D. T. J. Liley , D. B. Grayden , M. J. Cook , Brain 2018, 141, 2619. 30101347 10.1093/brain/awy210 PMC6136083

[29] A. Sherstinsky , Phys. D 2020, 404, 132306.

[30] W. K. Ojemann , B. H. Scheid , S. Mouchtaris , A. Lucas , J. J. LaRocque , C. Aguila , A. Ashourvan , L. Caciagli , K. A. Davis , E. C. Conrad , B. Litt , Brain Stimul. 2023, 16, 1709. 37979654 10.1016/j.brs.2023.11.005 PMC12961971

[31] R. G. Andrzejak , H. P. Zaveri , A. Schulze‐Bonhage , M. G. Leguia , W. C. Stacey , M. P. Richardson , L. Kuhlmann , K. Lehnertz , Epilepsia 2023, 64, S62. 36780237 10.1111/epi.17546 PMC10423299

[32] N. M. Gregg , T. P. Attia , M. Nasseri , B. Joseph , P. Karoly , J. Cui , R. E. Stirling , P. F. Viana , T. J. Richner , E. S. Nurse , A. Schulze‐Bonhage , M. J. Cook , G. A. Worrell , M. P. Richardson , D. R. Freestone , B. H. Brinkmann , Epilepsia 2023, 64, 1627. 37060170 10.1111/epi.17607 PMC10733995

[33] B. A. Malow , R. J. Bowes , X. Lin , Sleep 1997, 20, 1105. 9493919 10.1093/sleep/20.12.1105

[34] T. Proix , M. Baud , EBioMedicine 2021, 74. 10.1016/j.ebiom.2021.103698 PMC8605401 34800901

[35] A. G. Herzog , Seizure 2015, 28, 18. 25770028 10.1016/j.seizure.2015.02.024

[36] P. E. Schauwecker , BMC Neurosci. 2012, 13, 94. 22867059 10.1186/1471-2202-13-94 PMC3465215

[37] A. Vezzani , J. French , T. Bartfai , T. Z. Baram , Nat. Rev. Neurol. 2011, 7, 31. 21135885 10.1038/nrneurol.2010.178 PMC3378051

[38] D. N. Anderson , C. M. Charlebois , E. H. Smith , T. S. Davis , A. Y. Peters , B. J. Newman , A. M. Arain , K. S. Wilcox , C. R. Butson , J. D. Rolston , Brain 2023, 147, 521. 10.1093/brain/awad343 PMC10834245 37796038

[39] S. Chiang , A. N. Khambhati , T. K. Tcheng , A. P. Loftman , N. R. Hasulak , E. A. Mirro , M. J. Morrell , V. R. Rao , Brain 2024, 148, 521. 10.1093/brain/awae240 PMC11788193 39052924

[40] M. J. Cook , T. J. O'Brien , S. F. Berkovic , M. Murphy , A. Morokoff , G. Fabinyi , W. D'Souza , R. Yerra , J. Archer , L. Litewka , S. Hosking , P. Lightfoot , V. Ruedebusch , W. D. Sheffield , D. Snyder , K. Leyde , D. Himes , Lancet Neurol. 2013, 12, 563. 23642342 10.1016/S1474-4422(13)70075-9

[41] L. S. Remvig , J. Duun‐Henriksen , F. Fürbass , M. Hartmann , P. F. Viana , A. M. Kappel Overby , S. Weisdorf , M. P. Richardson , S. Beniczky , T. W. Kjaer , Clin. Neurophysiol. 2022, 142, 86. 35987094 10.1016/j.clinph.2022.07.504

[42] N. M. Gregg , V. Sladky , P. Nejedly , F. Mivalt , I. Kim , I. Balzekas , B. K. Sturges , C. Crowe , E. E. Patterson , J. J. Van Gompel , B. N. Lundstrom , K. Leyde , T. J. Denison , B. H. Brinkmann , V. Kremen , G. A. Worrell , Sci. Rep. 2021, 11, 24250. 34930926 10.1038/s41598-021-03555-7 PMC8688461

[43] S. N. Rønborg , R. Esteller , T. K. Tcheng , D. A. Greene , M. J. Morrell , T. W. Kjaer , S. A. Desai , Clinical Neurophysiology 2021, 132, 1209. 33931295 10.1016/j.clinph.2021.03.013

[44] V. S. Sohal , F. T. Sun , Neurosurg. Clin. 2011, 22, 481. 10.1016/j.nec.2011.07.007 21939847

[45] A. M. Fraser , H. L. Swinney , Phys. Rev. A Gen. Phys. 1986, 33, 1134. 9896728 10.1103/physreva.33.1134

[46] M. B. Kennel , R. Brown , H. D. Abarbanel , Phys. Rev. A 1992, 45, 3403. 9907388 10.1103/physreva.45.3403

