# Supplementary material for: Epileptiform Activity and Seizure Risk Follow Long‐Term Non‐Linear Attractor Dynamics
Source: Adv Sci (Weinh). 2025 Apr 7;12(23):2411829. doi: 10.1002/advs.202411829 (PMC12199362; doi:10.1002/advs.202411829)
Supplement: Supplementary file 1 — Supporting Information [file ADVS-12-2411829-s001.pdf]

ADVANCED SCIENCE
Open Access

Supporting Information

for *Adv. Sci.*, DOI 10.1002/advs.202411829

Epileptiform Activity and Seizure Risk Follow Long-Term Non-Linear Attractor Dynamics

Richard E Rosch, Brittany Scheid, Kathryn A Davis, Brian Litt and Arian Ashourvan**

Supplementary document for "Epileptiform activity and seizure risk follow long-term non-linear attractor dynamics"

Richard E Rosch[1,2,*], Brittany Scheid[3,4,*], Kathryn A Davis[5], Brian Litt[3,4,5], and Arian Ashourvan[6,†]

[1]Departments of Pediatrics and Neurology, Columbia University Irving Medical Center, New York, NY, USA
[2]Department of Basic and Clinical Neuroscience, Institute of Psychiatry, Psychology and Neuroscience, King's College London, London, UK
[3]Department of Bioengineering, School of Engineering and Applied Science, University of Pennsylvania
[4]Penn Center for Neuroengineering and Therapeutics, University of Pennsylvania
[5]Department of Neurology, Perelman School of Medicine, University of Pennsylvania, Philadelphia, PA, USA
[6]Department of Psychology, University of Kansas
[†]Correspondence to: Richard Rosch, Maurice Wohl Clinical Neuroscience Institute, 5 Cutcombe Road, London SE5 9RX. Email: richard@dynamic-brains.com, and Arian Ashourvan, 1415 Jayhawk Blvd, Lawrence, KS 66045, Email: ashourvan@ku.edu

March 20, 2025

Supplementary Figures

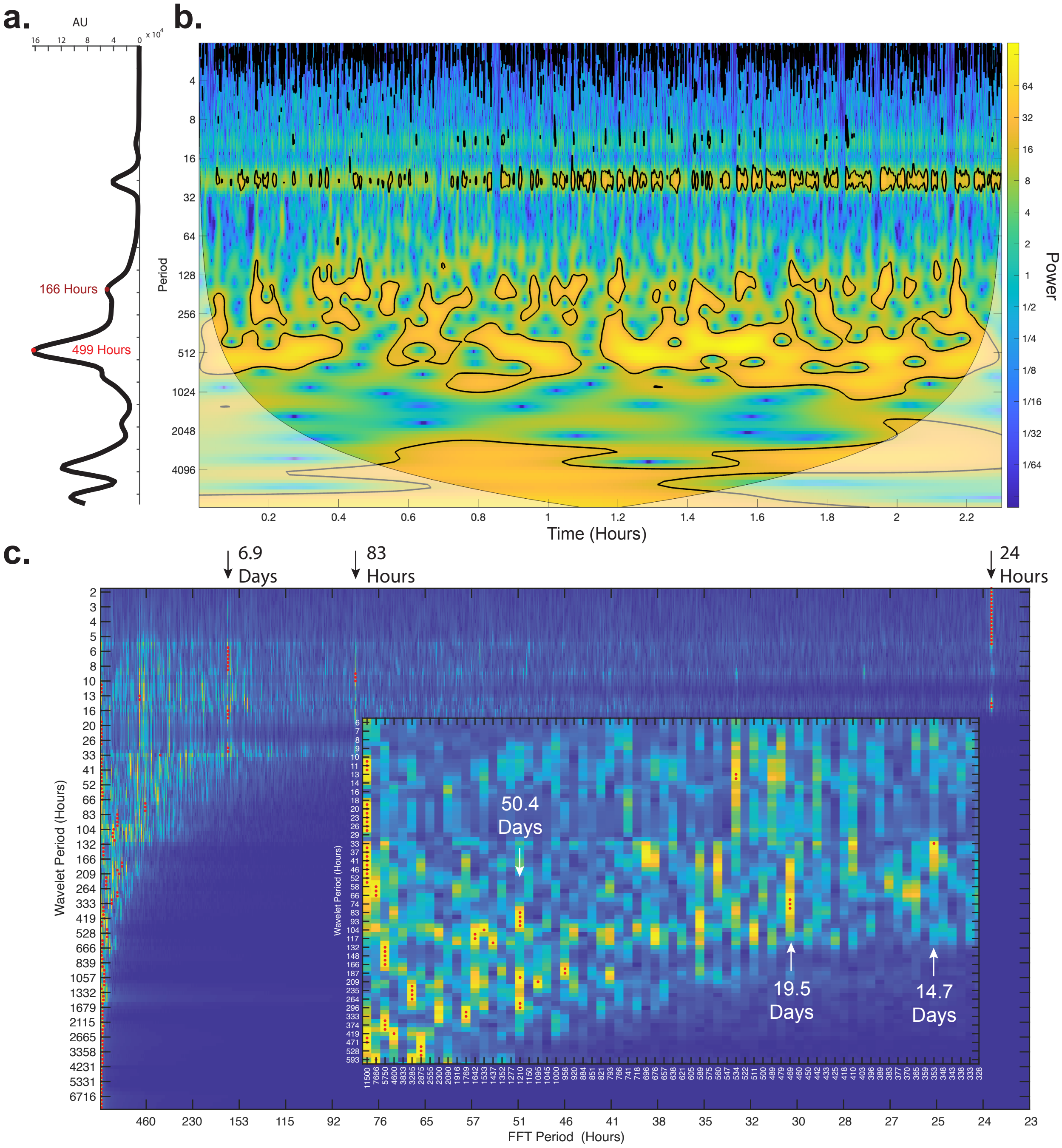

Figure S1: Identifying the slow multi-day peaks. The periodogram ***a.*** and wavelet ***b.*** decomposition of a sample patient's IEA-count time series. The periodogram in panel ***a*** is created by summing the power of the wavelet decomposition over all time points for each scale (i.e., period). The two identified multi-day peaks are indicated on the plot. ***c.*** Fast Fourier Transform (FFT) decomposition of wavelet time series for each scale. The red dots show the identified peak frequency. We highlighted the period of several dominant power amplitude-modulating frequencies with arrows.

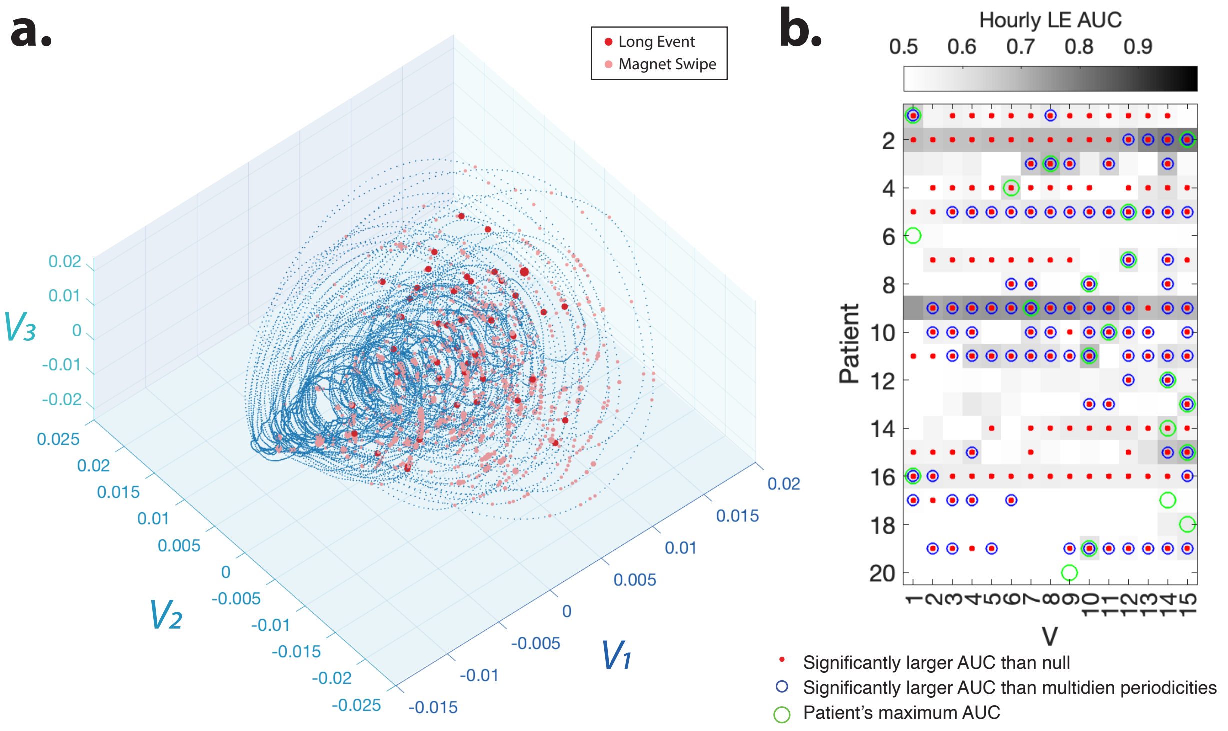

Figure S2: Delay-embedded IEA-count time series coordinates detect the seizure risk. ***a.*** Patient-labeled seizures (red dots) and device-labeled long IEA events (pink dots) overlap with the region of the manifold marked by the increased forcing in Figure 1e in the main text. ***b.*** The mean hourly area under the receiver operating characteristic curve (AUC) for detection of seizure risk (long IEA events) using different delay-embedding coordinates over 50 repetitions of the analysis (see Materials and Methods for classification details). Red dots show the mean AUC values that are significantly higher than those of the random null detection (two-sample $t-$test, $p < 0.05$, Bonferroni corrected for multiple comparisons across patients and coordinates. See Statistics section for more details on the null and permutation test). Blue circles show the mean AUC values that are significantly (two-sample $t-$test, $p < 0.05$, Bonferroni corrected for multiple comparisons) higher than the AUC values calculated from the two slow peak features (i.e., their amplitude and phase). Green circles show the maximum mean AUC across all coordinates for each patient.

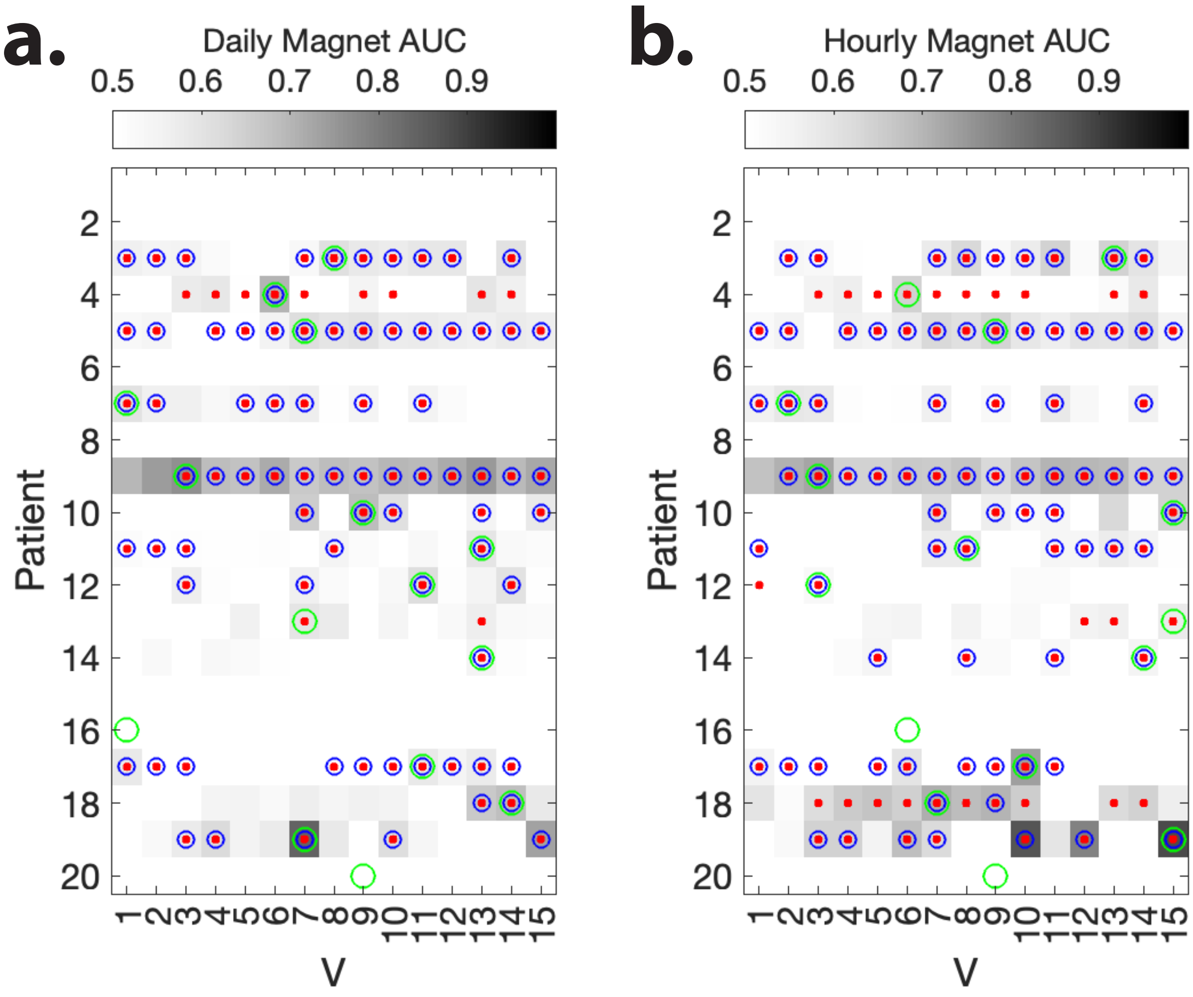

Figure S3: Delay-embedded coordinates of the IEA-count time series detect the patient-labeled seizure risk. The mean daily ***(a)*** and hourly ***(b)*** Area Under the Receiver Operating Characteristic Curve (AUC) for detection of patient-labeled seizure risk using different delay-embedding coordinates over 50 repetitions of the analysis (see Materials and Methods for classification details). Red dots show the mean AUC values that are significantly higher than those of the random null detection (two-sample $t-$test, $p < 0.05$, Bonferroni corrected for multiple comparisons across patients and coordinates. See Statistics section for more details on the null and permutation test). Blue circles show the mean AUC values that are significantly (two-sample $t-$test, $p < 0.05$, Bonferroni corrected for multiple comparisons) higher than the AUC values calculated from the two slow peak features (i.e., their amplitude and phase). Green circles show the maximum mean AUC across all coordinates for each patient.

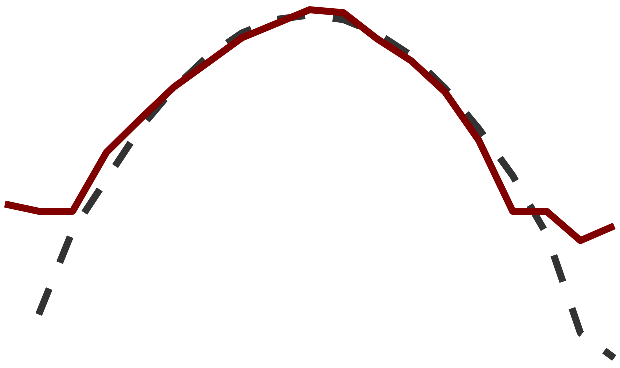

Figure S4: Sample patient's forcing statistics. The red curve shows the distribution of the forcing (V_{11}) in manuscript Figure 3. The black curve shows the Gaussian distribution of random variables with the same standard deviation as V_{11}. The distribution of the forcing exhibits a nearly symmetrical pattern with heavy tails, indicating the presence of infrequent and extreme forcing events.

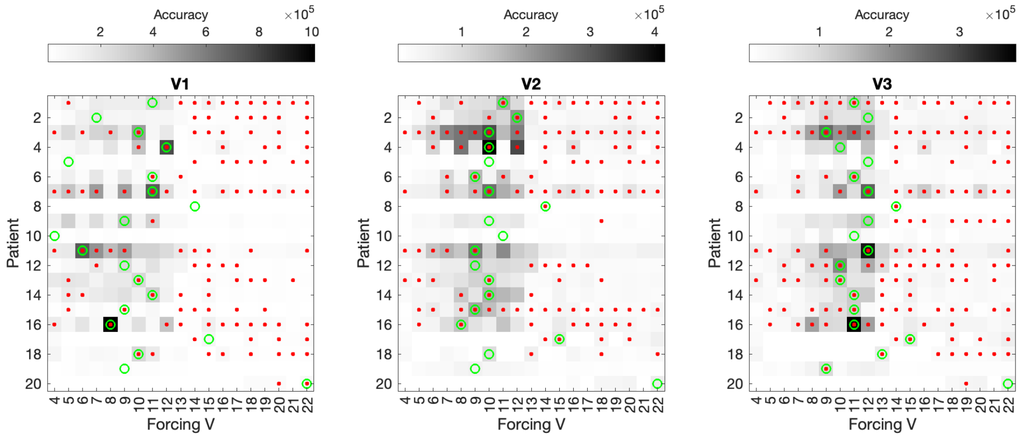

Figure S5: Accuracy of the Linear system of IEA delay-embedded coordinates' predicted trajectory *d*. The accuracy (the inverse of mean squared error) of the predicted first three leading coordinate (V_1, V_2, and V_3) time series for systems of different sizes with V_4 to V_{22} as forcing. Red dots indicate significantly higher accuracy than those of the future trajectory predictions of the phase-randomized IEA-count null time series ($p < 0.05$, two-tailed, $n = 50$ iterations, FDR corrected for multiple comparisons across patients and delay-embedded coordinates).

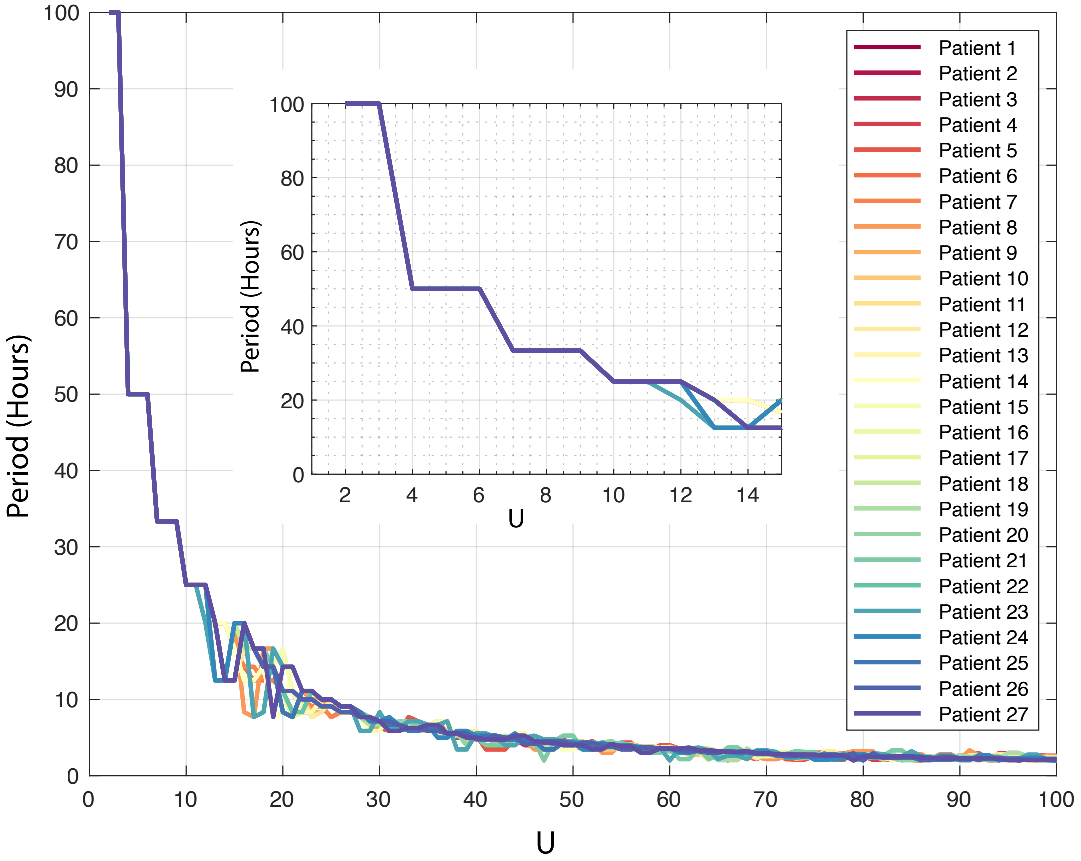

Figure S6: Peak periods of the U basis vectors The period of peak frequency identified for all U basis vectors identified using Fast Fourier Transform (FFT). Different Patients are color-coded. Inset shows the same plot for U_1 and U_{15} values.

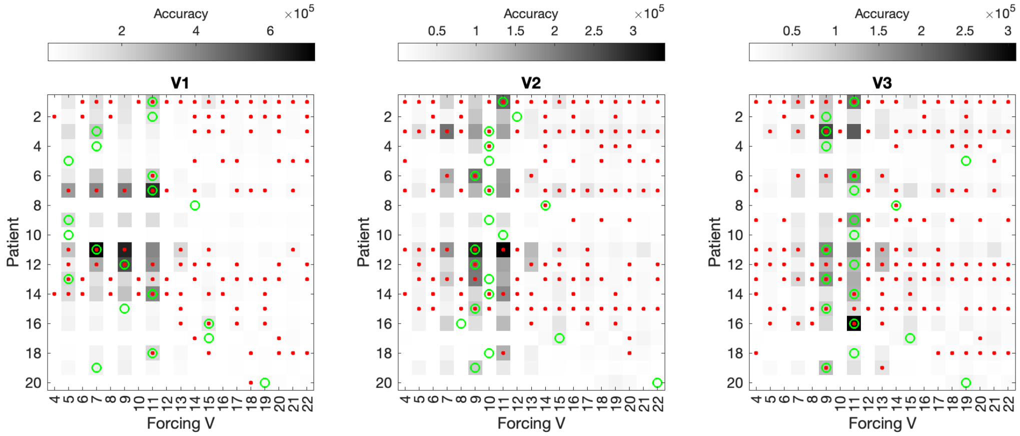

Figure S7: Accuracy of the Linear system of IEA delay-embedded coordinates' predicted trajectory using convolution-based forcing calculation *d*. The accuracy (the inverse of mean squared error) of the predicted first three leading coordinate (V_1, V_2, and V_3) time series for systems of different sizes with V_4 to V_{22} as forcing. The forcing was directly calculated from the IEA-count time series by convolution with forcing's corresponding basis vector (i.e., U_r column of U matrix). Interestingly, the accuracy of the systems with an odd number of dimensions is lower due to the inverted sign of the predicted time series (i.e., the predicted time series are flipped compared to the original delay-embedded coordinates' trajectories). Red dots indicate significantly higher accuracy than those of the future trajectory predictions of the phase-randomized IEA-count null time series ($p < 0.05$, two-tailed, $n = 50$ iterations, FDR corrected for multiple comparisons across patients and delay-embedded coordinates).

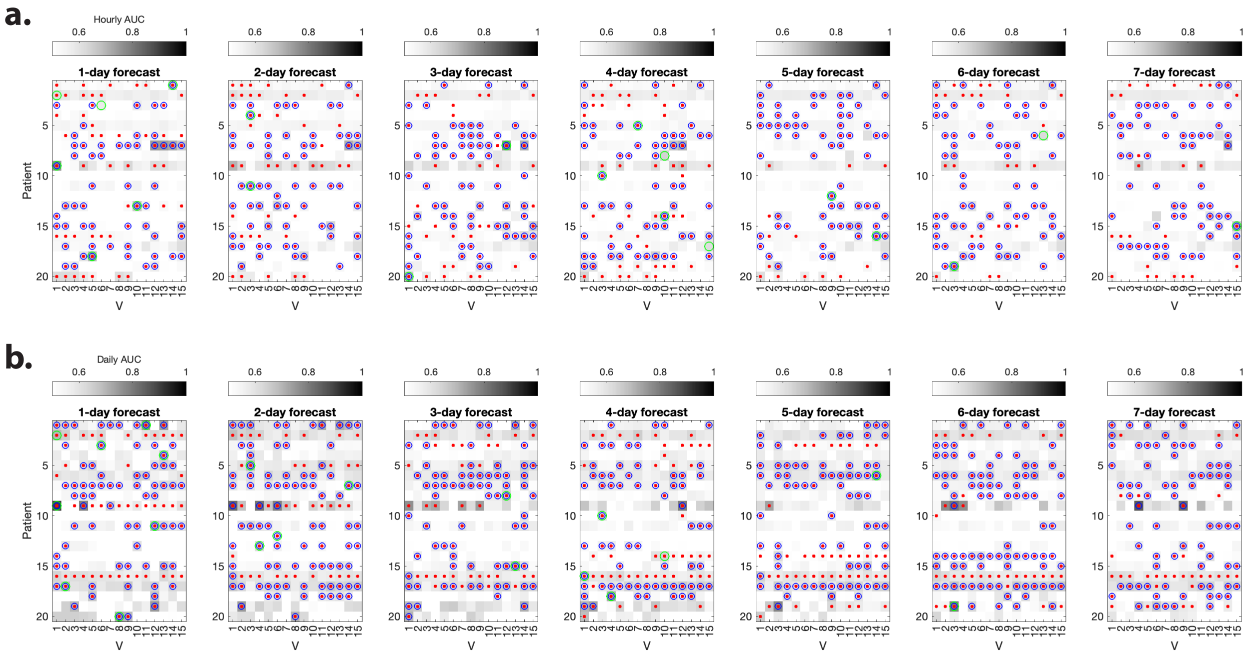

Figure S8: Delay-embedded coordinates of the IEA-count time series enable forecast of the seizure risk. The mean hourly ***(a)*** and daily ***(b)*** Area Under the Receiver Operating Characteristic Curve (AUC) for the forecast (1- to 7-days) of seizure risk (long IEA events) using different delay-embedding coordinates over 50 repetitions of the analysis (see Materials and Methods for classification details). Red dots show the mean AUC values that are significantly higher than those of the random null forecast (two-sample t−test, $p < 0.05$, Bonferroni corrected for multiple comparisons across patients and coordinates. See Statistics section for more details on the null and permutation test). Blue circles show the mean AUC values that are significantly (two-sample t−test, $p < 0.05$, Bonferroni corrected for multiple comparisons) higher than the AUC values calculated from the two slow peak features (i.e., their amplitude and phase). Green circles show the maximum mean AUC across all coordinates and all days for each patient.

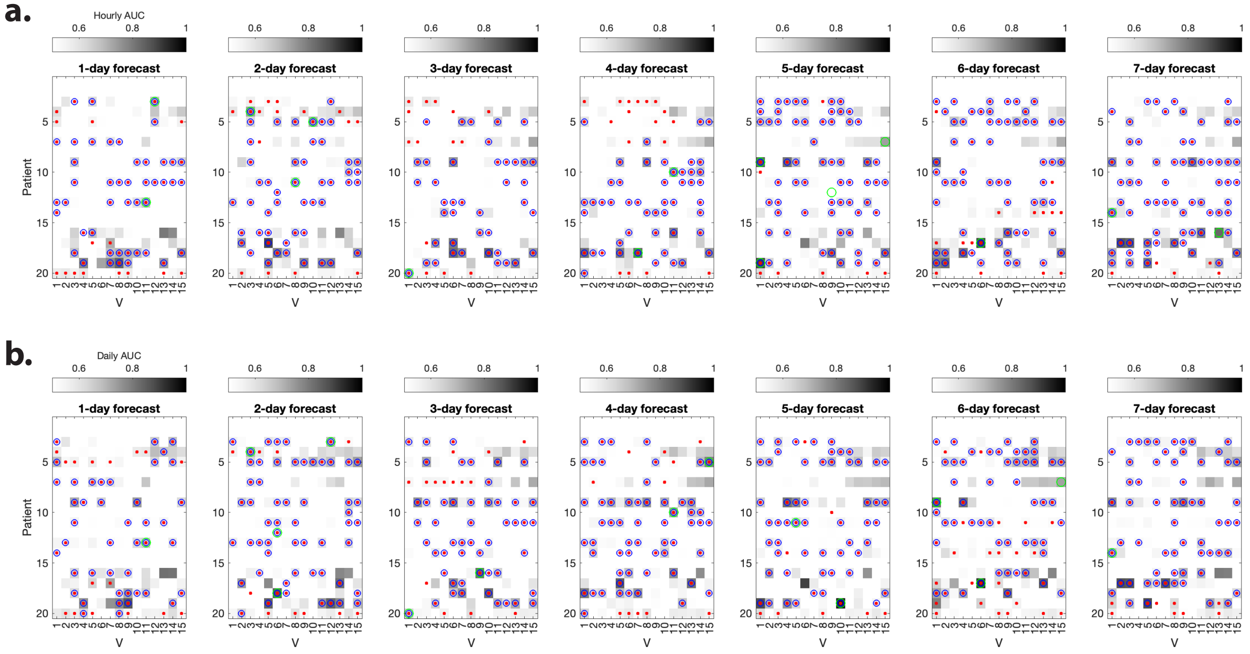

Figure S9: Delay-embedded coordinates of the IEA-count time series enable forecast of the patient-labeled seizure risk. The mean hourly ***(a)*** and daily ***(b)*** Area Under the Receiver Operating Characteristic Curve (AUC) for the forecast (1- to 7-days) of patient-labeled seizure risk using different delay-embedding coordinates over 50 repetitions of the analysis (see Materials and Methods for classification details). Red dots show the mean AUC values that are significantly higher than those of the random null forecast (two-sample $t-$test, $p < 0.05$, Bonferroni corrected for multiple comparisons across patients and coordinates. See Statistics section for more details on the null and permutation test). Blue circles show the mean AUC values that are significantly (two-sample $t-$test, $p < 0.05$, Bonferroni corrected for multiple comparisons) higher than the AUC values calculated from the two slow peak features (i.e., their amplitude and phase). Green circles show the maximum mean AUC across all coordinates and all days for each patient.

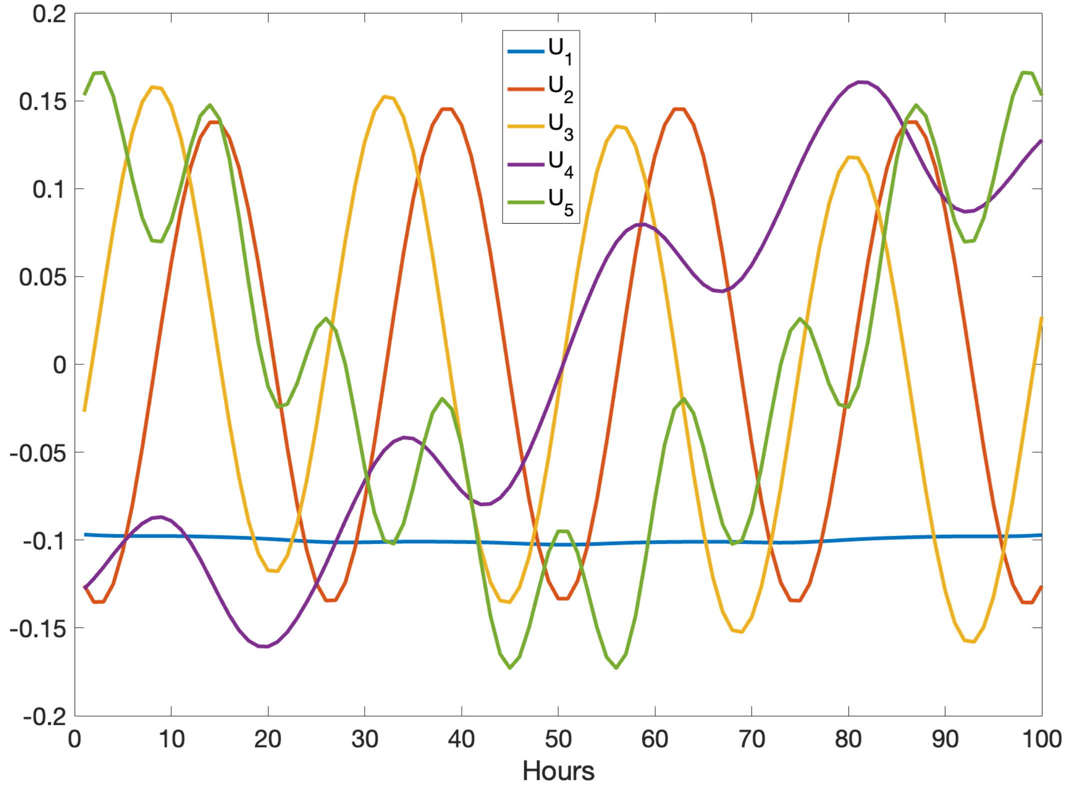

Figure S10: The U basis vectors 1 to 5 without low-pass (3-day) IEA-count time series filtering.
